# 5-Methyl-1,2,3,3a-tetra­hydro­benzo[*e*]pyrrolo­[2,1-*b*][1,3]oxazepin-10(5*H*)-one

**DOI:** 10.1107/S160053681102647X

**Published:** 2011-07-09

**Authors:** Yun-Zhou Jin, Rong-Hua Zhang, Da-Xu Fu, Yao-Kang Lv

**Affiliations:** a Chemistry Department, Tongji University, Shanghai 200092, People’s Republic of China

## Abstract

The asymmetric unit of the title compound, C_13_H_15_NO_2_, the main product of a photoreaction, contains two crystallographically independent mol­ecules. In both mol­ecules, the conformation of the seven-membered ring is twist sofa and that of the five-membered rings is envelope. In the crystal, mol­ecules are linked by weak inter­molecular C—H⋯O hydrogen bonds.

## Related literature

For general background to asymmetric photochemical reactions, see: Aubert *et al.* (2000 ▶); Gratzel (2001 ▶); Korzeniewski & Zoladz (2001 ▶). For photo-induced cyclizations, see Griesbeck *et al.* (2002 ▶); Henz *et al.* (1995 ▶); For related structures, see: Basarić *et al.* (2008 ▶); Griesbeck *et al.* (1997 ▶, 1999 ▶); Jin *et al.* (2011*a* ▶,*b* ▶).
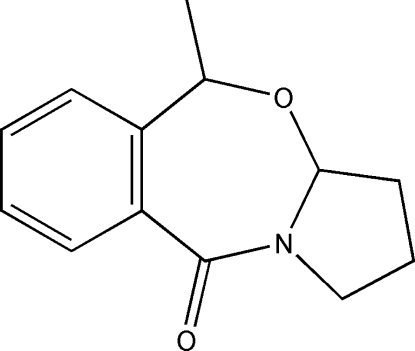


## Experimental

### 

#### Crystal data

C_13_H_15_NO_2_
*M* *_r_* = 217.26
Orthorhombic, 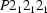

*a* = 10.410 (4) Å
*b* = 12.688 (5) Å
*c* = 17.124 (7) Å
*V* = 2261.8 (15) Å^3^
*Z* = 8
Mo *K*α radiation
μ = 0.09 mm^−1^
*T* = 296 K
0.23 × 0.20 × 0.18 mm

#### Data collection

Rigaku SCXmini diffractometer
Absorption correction: multi-scan (*SADABS*; Sheldrick, 1996 ▶) *T*_min_ = 0.97, *T*_max_ = 0.99
19530 measured reflections
2918 independent reflections
2555 reflections with *I* > 2σ(*I*)
*R*_int_ = 0.074

#### Refinement

*R*[*F* ^2^ > 2σ(*F* ^2^)] = 0.048
*wR*(*F* ^2^) = 0.108
*S* = 0.99
2918 reflections
291 parameters
H-atom parameters constrained
Δρ_max_ = 0.42 e Å^−3^
Δρ_min_ = −0.39 e Å^−3^

### 

Data collection: *CrystalClear* (Rigaku, 2005 ▶); cell refinement: *CrystalClear*; data reduction: *CrystalClear*; program(s) used to solve structure: *SHELXS97* (Sheldrick, 2008 ▶); program(s) used to refine structure: *SHELXL97* (Sheldrick, 2008 ▶); molecular graphics: *DIAMOND* (Brandenburg & Putz, 2005 ▶); software used to prepare material for publication: *SHELXL97*.

## Supplementary Material

Crystal structure: contains datablock(s) I, global. DOI: 10.1107/S160053681102647X/ff2019sup1.cif

Structure factors: contains datablock(s) I. DOI: 10.1107/S160053681102647X/ff2019Isup2.hkl

Supplementary material file. DOI: 10.1107/S160053681102647X/ff2019Isup3.cml

Additional supplementary materials:  crystallographic information; 3D view; checkCIF report

## Figures and Tables

**Table 1:** Hydrogen-bond geometry (Å, °)

*D*—H⋯*A*	*D*—H	H⋯*A*	*D*⋯*A*	*D*—H⋯*A*
C4—H4*A*⋯O1^i^	0.93	2.54	3.293 (4)	139
C16—H16*A*⋯O4^ii^	0.93	2.58	3.243 (3)	129

Symmetry codes: (i) 
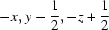
; (ii) 
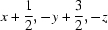
.

**Fig. 1.**
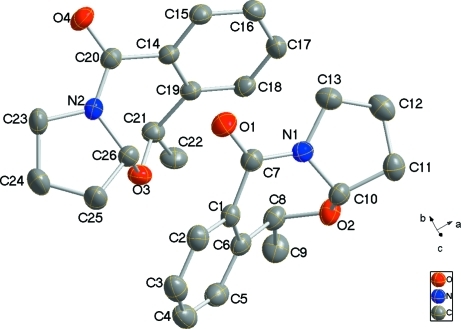
The molecular structure of the title compound with displacement ellipsoids drawn at the 30% probability level. H atoms are omitted for clarity.

**Fig. 2.**
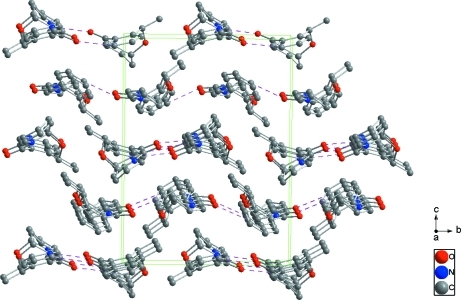
Packing diagram showing the C—H···O interactions.

## supplementary crystallographic information

### Comment

In modern organic chemistry preparative organic photochemistry is an important tool to synthesize the compounds in one step which cannot be gained in common reactions. (Aubert *et al.* 2000; Gratzel, 2001; Korzeniewski & Zoladz, 2001). Benzophenone acylamide derivatives can form the seven-membered ring through the intramolecular photoinduced decarboxylation and cyclization (Griesbeck *et al.*, 2002; Henz *et al.*,1995). Recently, we have reported two seven-membered ring compounds prepared by photochemical reaction (Jin *et al.*, 2011*a*; Jin *et al.*, 2011*b*).

We report herein the crystal structure and synthesis of the title compound. Single crystal *X*–ray analysis revealed that the title compound crystallizes in orthorhombic, chiral space group *P*2_1_2_1_2_1_. The asymmetric unit contains two crystallographically independent molecules. As shown in Fig.1, the two molecules, which have the opposite absolute configuration, have the same molecular formula containing one seven-membered ring, one five-membered ring and one six-membered ring. The enantiomers have slightly different bond lengths and bond angles and atoms C8, C10, C21, C26 are chiral centers. The crystal packing exhibits weak intermolecular C—H···O hydrogen bonds (Fig. 2).

### Experimental

The title compound, C_13_H_15_NO_2_, was the main product from the photoreaction of (*S*)-1-(2-acetylbenzoyl) pyrrolidine-2-carboxylic acid under N_2_ for 10 h. The compound was purified by flash column chromatography (silica gel column, petroleum ether/ethyl acetate=6/1). Colourless crystals for the X-ray crystallographic studies were gained by slow evaporation of a dichloromethane solution.

### Refinement

The structure was solved by direct methods and expanded with difference Fourier techniques. All non-hydrogen atoms were refined anisotropically by the full matrix least-squares on the F^2^. The hydrogen atoms attached to carbon atoms were located by geometrical calculation using a riding model [*U*_iso_(H) = 1.2*U*_eq_(C)].

### Crystal data

**Table:**

C_13_H_15_NO_2_	*F*(000) = 928
*M**_r_* = 217.26	*D*_x_ = 1.276 Mg m^−^^3^
Orthorhombic, *P*2_1_2_1_2_1_	Mo *K*α radiation, λ = 0.71073 Å
Hall symbol: P 2ac 2ab	Cell parameters from 7042 reflections
*a* = 10.410 (4) Å	θ = 2.3–27.5°
*b* = 12.688 (5) Å	µ = 0.09 mm^−^^1^
*c* = 17.124 (7) Å	*T* = 296 K
*V* = 2261.8 (15) Å^3^	Prism, colourless
*Z* = 8	0.23 × 0.20 × 0.18 mm

### Data collection

**Table:**

Rigaku SCXmini diffractometer	2918 independent reflections
Radiation source: fine-focus sealed tube	2555 reflections with *I* > 2σ(*I*)
graphite	*R*_int_ = 0.074
ω scans	θ_max_ = 27.5°, θ_min_ = 3.2°
Absorption correction: multi-scan (*SADABS*; Sheldrick, 1996)	*h* = −13→13
*T*_min_ = 0.97, *T*_max_ = 0.99	*k* = −16→11
19530 measured reflections	*l* = −22→22

### Refinement

**Table:**

Refinement on *F*^2^	Secondary atom site location: difference Fourier map
Least-squares matrix: full	Hydrogen site location: inferred from neighbouring sites
*R*[*F*^2^ > 2σ(*F*^2^)] = 0.048	H-atom parameters constrained
*wR*(*F*^2^) = 0.108	*w* = 1/[σ^2^(*F*_o_^2^) + (0.0595*P*)^2^] where *P* = (*F*_o_^2^ + 2*F*_c_^2^)/3
*S* = 0.99	(Δ/σ)_max_ < 0.001
2918 reflections	Δρ_max_ = 0.42 e Å^−^^3^
291 parameters	Δρ_min_ = −0.39 e Å^−^^3^
0 restraints	Extinction correction: *SHELXL97* (Sheldrick, 2008), Fc^*^=kFc[1+0.001xFc^2^λ^3^/sin(2θ)]^-1/4^
Primary atom site location: structure-invariant direct methods	Extinction coefficient: 0.026 (3)

### Special details

**Table:**

Geometry. All e.s.d.'s (except the e.s.d. in the dihedral angle between two l.s. planes) are estimated using the full covariance matrix. The cell e.s.d.'s are taken into account individually in the estimation of e.s.d.'s in distances, angles and torsion angles; correlations between e.s.d.'s in cell parameters are only used when they are defined by crystal symmetry. An approximate (isotropic) treatment of cell e.s.d.'s is used for estimating e.s.d.'s involving l.s. planes.
Refinement. Refinement of *F*^2^ against ALL reflections. The weighted *R*-factor *wR* and goodness of fit *S* are based on *F*^2^, conventional *R*-factors *R* are based on *F*, with *F* set to zero for negative *F*^2^. The threshold expression of *F*^2^ > σ(*F*^2^) is used only for calculating *R*-factors(gt) *etc*. and is not relevant to the choice of reflections for refinement. *R*-factors based on *F*^2^ are statistically about twice as large as those based on *F*, and *R*- factors based on ALL data will be even larger.

### Fractional atomic coordinates and isotropic or equivalent isotropic displacement parameters (Å^2^)

**Table:**

	*x*	*y*	*z*	*U*_iso_*/*U*_eq_	
O1	0.23788 (19)	0.54319 (13)	0.26755 (12)	0.0777 (6)	
O2	0.37121 (17)	0.22737 (13)	0.22168 (10)	0.0620 (5)	
O3	0.03656 (17)	0.37491 (12)	0.02903 (10)	0.0593 (4)	
O4	0.09595 (19)	0.70473 (12)	−0.01621 (11)	0.0678 (5)	
N1	0.37387 (19)	0.40475 (15)	0.26863 (11)	0.0521 (5)	
N2	0.01054 (19)	0.56105 (15)	0.04280 (11)	0.0522 (5)	
C1	0.1473 (2)	0.37156 (18)	0.28097 (13)	0.0507 (5)	
C2	0.0459 (3)	0.3994 (2)	0.33027 (14)	0.0626 (6)	
H2A	0.0492	0.4629	0.3573	0.075*	
C3	−0.0590 (3)	0.3341 (2)	0.33938 (17)	0.0734 (8)	
H3A	−0.1249	0.3524	0.3734	0.088*	
C4	−0.0649 (3)	0.2418 (3)	0.29778 (17)	0.0772 (8)	
H4A	−0.1361	0.1980	0.3029	0.093*	
C5	0.0341 (3)	0.2136 (2)	0.24836 (16)	0.0674 (7)	
H5A	0.0281	0.1509	0.2204	0.081*	
C6	0.1429 (2)	0.27645 (18)	0.23933 (13)	0.0525 (5)	
C7	0.2558 (2)	0.44788 (18)	0.27247 (13)	0.0538 (5)	
C8	0.2520 (3)	0.24980 (19)	0.18309 (14)	0.0583 (6)	
H8A	0.2657	0.3111	0.1493	0.070*	
C9	0.2278 (3)	0.1548 (2)	0.13068 (18)	0.0870 (10)	
H9A	0.3007	0.1439	0.0973	0.104*	
H9B	0.1529	0.1673	0.0993	0.104*	
H9C	0.2145	0.0933	0.1624	0.104*	
C10	0.4018 (2)	0.29467 (18)	0.28521 (14)	0.0535 (6)	
H10A	0.3571	0.2718	0.3326	0.064*	
C11	0.5462 (3)	0.2946 (2)	0.29797 (15)	0.0670 (7)	
H11A	0.5840	0.2288	0.2806	0.080*	
H11B	0.5669	0.3052	0.3526	0.080*	
C12	0.5938 (3)	0.3866 (2)	0.24849 (17)	0.0748 (8)	
H12A	0.6746	0.4135	0.2684	0.090*	
H12B	0.6056	0.3653	0.1946	0.090*	
C13	0.4909 (3)	0.4675 (2)	0.25502 (16)	0.0635 (6)	
H13A	0.5073	0.5149	0.2983	0.076*	
H13B	0.4839	0.5083	0.2073	0.076*	
C14	0.2354 (2)	0.55960 (15)	0.01113 (11)	0.0448 (5)	
C15	0.3450 (2)	0.61538 (18)	0.03210 (13)	0.0531 (6)	
H15A	0.3389	0.6869	0.0435	0.064*	
C16	0.4622 (3)	0.56595 (19)	0.03615 (15)	0.0590 (6)	
H16A	0.5352	0.6035	0.0506	0.071*	
C17	0.4708 (2)	0.4595 (2)	0.01856 (14)	0.0591 (6)	
H17A	0.5496	0.4252	0.0221	0.071*	
C18	0.3631 (2)	0.40425 (18)	−0.00418 (14)	0.0536 (5)	
H18A	0.3705	0.3332	−0.0166	0.064*	
C19	0.2441 (2)	0.45249 (16)	−0.00882 (12)	0.0449 (5)	
C20	0.1086 (2)	0.61564 (17)	0.01078 (13)	0.0494 (5)	
C21	0.1226 (2)	0.39619 (18)	−0.03595 (14)	0.0540 (6)	
H21A	0.0775	0.4439	−0.0717	0.065*	
C22	0.1428 (3)	0.2944 (2)	−0.07828 (17)	0.0746 (8)	
H22A	0.0612	0.2663	−0.0940	0.090*	
H22B	0.1851	0.2452	−0.0444	0.090*	
H22C	0.1950	0.3066	−0.1236	0.090*	
C23	−0.1212 (2)	0.6019 (2)	0.04741 (17)	0.0662 (7)	
H23A	−0.1548	0.6176	−0.0041	0.079*	
H23B	−0.1251	0.6650	0.0793	0.079*	
C24	−0.1937 (3)	0.5125 (2)	0.0848 (2)	0.0869 (10)	
H24A	−0.2595	0.5395	0.1196	0.104*	
H24B	−0.2344	0.4691	0.0453	0.104*	
C25	−0.0971 (3)	0.4498 (2)	0.12943 (16)	0.0693 (7)	
H25A	−0.1232	0.3766	0.1332	0.083*	
H25B	−0.0862	0.4779	0.1817	0.083*	
C26	0.0263 (2)	0.46025 (18)	0.08272 (13)	0.0533 (5)	
H26A	0.1009	0.4622	0.1176	0.064*	

### Atomic displacement parameters (Å^2^)

**Table:**

	*U*^11^	*U*^22^	*U*^33^	*U*^12^	*U*^13^	*U*^23^
O1	0.0809 (13)	0.0451 (10)	0.1070 (15)	0.0058 (9)	−0.0035 (12)	−0.0016 (10)
O2	0.0678 (11)	0.0560 (9)	0.0622 (10)	0.0146 (8)	−0.0134 (9)	−0.0095 (8)
O3	0.0592 (10)	0.0500 (9)	0.0688 (10)	−0.0100 (8)	0.0036 (8)	0.0016 (7)
O4	0.0801 (12)	0.0481 (9)	0.0753 (11)	0.0147 (8)	0.0024 (10)	0.0140 (8)
N1	0.0540 (11)	0.0476 (10)	0.0548 (11)	−0.0012 (8)	−0.0003 (9)	0.0018 (8)
N2	0.0474 (11)	0.0525 (11)	0.0567 (11)	0.0062 (8)	−0.0044 (8)	0.0079 (9)
C1	0.0525 (13)	0.0529 (12)	0.0467 (11)	0.0061 (10)	−0.0077 (10)	0.0023 (10)
C2	0.0630 (16)	0.0695 (16)	0.0552 (13)	0.0061 (13)	−0.0069 (12)	−0.0021 (12)
C3	0.0585 (17)	0.097 (2)	0.0652 (17)	−0.0001 (16)	−0.0028 (13)	0.0068 (15)
C4	0.0639 (17)	0.091 (2)	0.0769 (19)	−0.0153 (16)	−0.0126 (15)	0.0127 (16)
C5	0.0754 (19)	0.0584 (14)	0.0684 (15)	−0.0086 (13)	−0.0205 (14)	0.0023 (12)
C6	0.0608 (14)	0.0487 (12)	0.0480 (12)	0.0023 (10)	−0.0131 (10)	0.0027 (9)
C7	0.0616 (15)	0.0482 (13)	0.0516 (12)	0.0015 (11)	−0.0053 (11)	−0.0029 (10)
C8	0.0725 (17)	0.0528 (13)	0.0495 (12)	0.0060 (12)	−0.0113 (12)	−0.0018 (9)
C9	0.106 (3)	0.0815 (19)	0.0736 (18)	0.004 (2)	−0.0170 (18)	−0.0301 (15)
C10	0.0611 (14)	0.0525 (13)	0.0469 (12)	0.0028 (11)	−0.0067 (11)	0.0017 (10)
C11	0.0612 (16)	0.0827 (19)	0.0571 (15)	0.0086 (14)	−0.0092 (12)	−0.0003 (13)
C12	0.0594 (16)	0.086 (2)	0.0786 (18)	−0.0076 (15)	0.0072 (14)	−0.0154 (16)
C13	0.0668 (16)	0.0638 (15)	0.0599 (13)	−0.0108 (12)	0.0080 (12)	−0.0059 (11)
C14	0.0531 (12)	0.0396 (10)	0.0418 (10)	−0.0011 (9)	−0.0011 (9)	0.0024 (8)
C15	0.0600 (15)	0.0437 (12)	0.0556 (13)	−0.0056 (10)	−0.0052 (11)	0.0003 (10)
C16	0.0544 (14)	0.0601 (15)	0.0626 (14)	−0.0112 (12)	−0.0070 (12)	0.0044 (11)
C17	0.0470 (13)	0.0639 (14)	0.0665 (14)	0.0035 (12)	0.0003 (11)	0.0066 (12)
C18	0.0600 (14)	0.0414 (11)	0.0593 (13)	0.0031 (10)	0.0024 (11)	0.0002 (10)
C19	0.0501 (12)	0.0407 (10)	0.0441 (10)	−0.0034 (9)	−0.0009 (9)	0.0014 (8)
C20	0.0565 (13)	0.0462 (12)	0.0454 (11)	0.0019 (10)	−0.0035 (10)	0.0006 (9)
C21	0.0592 (14)	0.0493 (12)	0.0536 (13)	−0.0088 (11)	−0.0052 (11)	−0.0016 (10)
C22	0.091 (2)	0.0621 (16)	0.0712 (17)	−0.0203 (15)	−0.0019 (15)	−0.0138 (13)
C23	0.0501 (14)	0.0775 (17)	0.0710 (16)	0.0133 (13)	−0.0096 (12)	0.0073 (14)
C24	0.0487 (16)	0.103 (2)	0.109 (2)	0.0010 (16)	0.0019 (17)	0.0183 (19)
C25	0.0567 (16)	0.0806 (18)	0.0707 (16)	0.0025 (14)	0.0090 (13)	0.0142 (15)
C26	0.0525 (13)	0.0550 (13)	0.0525 (12)	−0.0013 (11)	−0.0036 (10)	0.0074 (10)

### Geometric parameters (Å, °)

**Table:**

O1—C7	1.226 (3)	C11—H11B	0.9700
O2—C10	1.419 (3)	C12—C13	1.488 (4)
O2—C8	1.434 (3)	C12—H12A	0.9700
O3—C26	1.424 (3)	C12—H12B	0.9700
O3—C21	1.453 (3)	C13—H13A	0.9700
O4—C20	1.228 (3)	C13—H13B	0.9700
N1—C7	1.347 (3)	C14—C15	1.390 (3)
N1—C10	1.455 (3)	C14—C19	1.404 (3)
N1—C13	1.474 (3)	C14—C20	1.499 (3)
N2—C20	1.350 (3)	C15—C16	1.374 (3)
N2—C26	1.459 (3)	C15—H15A	0.9300
N2—C23	1.468 (3)	C16—C17	1.387 (3)
C1—C2	1.397 (3)	C16—H16A	0.9300
C1—C6	1.402 (3)	C17—C18	1.379 (3)
C1—C7	1.495 (3)	C17—H17A	0.9300
C2—C3	1.380 (4)	C18—C19	1.384 (3)
C2—H2A	0.9300	C18—H18A	0.9300
C3—C4	1.372 (4)	C19—C21	1.526 (3)
C3—H3A	0.9300	C21—C22	1.495 (3)
C4—C5	1.381 (4)	C21—H21A	0.9800
C4—H4A	0.9300	C22—H22A	0.9600
C5—C6	1.394 (4)	C22—H22B	0.9600
C5—H5A	0.9300	C22—H22C	0.9600
C6—C8	1.527 (3)	C23—C24	1.506 (4)
C8—C9	1.524 (3)	C23—H23A	0.9700
C8—H8A	0.9800	C23—H23B	0.9700
C9—H9A	0.9600	C24—C25	1.492 (4)
C9—H9B	0.9600	C24—H24A	0.9700
C9—H9C	0.9600	C24—H24B	0.9700
C10—C11	1.519 (4)	C25—C26	1.519 (3)
C10—H10A	0.9800	C25—H25A	0.9700
C11—C12	1.525 (4)	C25—H25B	0.9700
C11—H11A	0.9700	C26—H26A	0.9800
			
C10—O2—C8	115.35 (17)	C12—C13—H13A	111.0
C26—O3—C21	113.53 (16)	N1—C13—H13B	111.0
C7—N1—C10	124.3 (2)	C12—C13—H13B	111.0
C7—N1—C13	122.85 (19)	H13A—C13—H13B	109.0
C10—N1—C13	112.6 (2)	C15—C14—C19	120.2 (2)
C20—N2—C26	123.71 (19)	C15—C14—C20	118.83 (19)
C20—N2—C23	123.17 (19)	C19—C14—C20	120.99 (19)
C26—N2—C23	112.89 (19)	C16—C15—C14	120.7 (2)
C2—C1—C6	120.1 (2)	C16—C15—H15A	119.7
C2—C1—C7	117.7 (2)	C14—C15—H15A	119.7
C6—C1—C7	122.2 (2)	C15—C16—C17	119.4 (2)
C3—C2—C1	120.9 (2)	C15—C16—H16A	120.3
C3—C2—H2A	119.5	C17—C16—H16A	120.3
C1—C2—H2A	119.5	C18—C17—C16	120.3 (2)
C4—C3—C2	119.3 (3)	C18—C17—H17A	119.9
C4—C3—H3A	120.3	C16—C17—H17A	119.9
C2—C3—H3A	120.3	C17—C18—C19	121.3 (2)
C3—C4—C5	120.4 (3)	C17—C18—H18A	119.4
C3—C4—H4A	119.8	C19—C18—H18A	119.4
C5—C4—H4A	119.8	C18—C19—C14	118.16 (19)
C4—C5—C6	121.7 (3)	C18—C19—C21	123.6 (2)
C4—C5—H5A	119.1	C14—C19—C21	118.27 (19)
C6—C5—H5A	119.1	O4—C20—N2	122.9 (2)
C5—C6—C1	117.5 (2)	O4—C20—C14	122.2 (2)
C5—C6—C8	123.2 (2)	N2—C20—C14	114.88 (18)
C1—C6—C8	119.1 (2)	O3—C21—C22	107.30 (19)
O1—C7—N1	122.4 (2)	O3—C21—C19	111.39 (18)
O1—C7—C1	122.0 (2)	C22—C21—C19	115.8 (2)
N1—C7—C1	115.5 (2)	O3—C21—H21A	107.3
O2—C8—C9	104.9 (2)	C22—C21—H21A	107.3
O2—C8—C6	113.38 (18)	C19—C21—H21A	107.3
C9—C8—C6	115.0 (2)	C21—C22—H22A	109.5
O2—C8—H8A	107.7	C21—C22—H22B	109.5
C9—C8—H8A	107.7	H22A—C22—H22B	109.5
C6—C8—H8A	107.7	C21—C22—H22C	109.5
C8—C9—H9A	109.5	H22A—C22—H22C	109.5
C8—C9—H9B	109.5	H22B—C22—H22C	109.5
H9A—C9—H9B	109.5	N2—C23—C24	103.0 (2)
C8—C9—H9C	109.5	N2—C23—H23A	111.2
H9A—C9—H9C	109.5	C24—C23—H23A	111.2
H9B—C9—H9C	109.5	N2—C23—H23B	111.2
O2—C10—N1	112.53 (18)	C24—C23—H23B	111.2
O2—C10—C11	109.4 (2)	H23A—C23—H23B	109.1
N1—C10—C11	103.1 (2)	C25—C24—C23	106.3 (2)
O2—C10—H10A	110.5	C25—C24—H24A	110.5
N1—C10—H10A	110.5	C23—C24—H24A	110.5
C11—C10—H10A	110.5	C25—C24—H24B	110.5
C10—C11—C12	104.0 (2)	C23—C24—H24B	110.5
C10—C11—H11A	111.0	H24A—C24—H24B	108.7
C12—C11—H11A	111.0	C24—C25—C26	104.7 (2)
C10—C11—H11B	111.0	C24—C25—H25A	110.8
C12—C11—H11B	111.0	C26—C25—H25A	110.8
H11A—C11—H11B	109.0	C24—C25—H25B	110.8
C13—C12—C11	104.6 (2)	C26—C25—H25B	110.8
C13—C12—H12A	110.8	H25A—C25—H25B	108.9
C11—C12—H12A	110.8	O3—C26—N2	111.86 (18)
C13—C12—H12B	110.8	O3—C26—C25	109.7 (2)
C11—C12—H12B	110.8	N2—C26—C25	103.2 (2)
H12A—C12—H12B	108.9	O3—C26—H26A	110.6
N1—C13—C12	103.6 (2)	N2—C26—H26A	110.6
N1—C13—H13A	111.0	C25—C26—H26A	110.6
			
C6—C1—C2—C3	0.6 (3)	C19—C14—C15—C16	−2.2 (3)
C7—C1—C2—C3	178.3 (2)	C20—C14—C15—C16	177.4 (2)
C1—C2—C3—C4	−1.7 (4)	C14—C15—C16—C17	0.4 (4)
C2—C3—C4—C5	1.2 (4)	C15—C16—C17—C18	1.2 (4)
C3—C4—C5—C6	0.4 (4)	C16—C17—C18—C19	−1.1 (4)
C4—C5—C6—C1	−1.6 (4)	C17—C18—C19—C14	−0.7 (3)
C4—C5—C6—C8	−177.7 (2)	C17—C18—C19—C21	178.1 (2)
C2—C1—C6—C5	1.0 (3)	C15—C14—C19—C18	2.3 (3)
C7—C1—C6—C5	−176.6 (2)	C20—C14—C19—C18	−177.32 (19)
C2—C1—C6—C8	177.4 (2)	C15—C14—C19—C21	−176.5 (2)
C7—C1—C6—C8	−0.3 (3)	C20—C14—C19—C21	3.9 (3)
C10—N1—C7—O1	170.8 (2)	C26—N2—C20—O4	−173.0 (2)
C13—N1—C7—O1	−2.6 (3)	C23—N2—C20—O4	1.0 (4)
C10—N1—C7—C1	−10.8 (3)	C26—N2—C20—C14	6.5 (3)
C13—N1—C7—C1	175.8 (2)	C23—N2—C20—C14	−179.5 (2)
C2—C1—C7—O1	−40.6 (3)	C15—C14—C20—O4	43.3 (3)
C6—C1—C7—O1	137.2 (2)	C19—C14—C20—O4	−137.1 (2)
C2—C1—C7—N1	141.0 (2)	C15—C14—C20—N2	−136.3 (2)
C6—C1—C7—N1	−41.2 (3)	C19—C14—C20—N2	43.3 (3)
C10—O2—C8—C9	−168.0 (2)	C26—O3—C21—C22	169.6 (2)
C10—O2—C8—C6	−41.7 (3)	C26—O3—C21—C19	41.9 (3)
C5—C6—C8—O2	−114.7 (2)	C18—C19—C21—O3	106.7 (2)
C1—C6—C8—O2	69.2 (3)	C14—C19—C21—O3	−74.5 (2)
C5—C6—C8—C9	6.1 (3)	C18—C19—C21—C22	−16.2 (3)
C1—C6—C8—C9	−170.0 (2)	C14—C19—C21—C22	162.5 (2)
C8—O2—C10—N1	−42.3 (3)	C20—N2—C23—C24	178.4 (2)
C8—O2—C10—C11	−156.2 (2)	C26—N2—C23—C24	−7.0 (3)
C7—N1—C10—O2	79.5 (3)	N2—C23—C24—C25	24.1 (3)
C13—N1—C10—O2	−106.5 (2)	C23—C24—C25—C26	−32.2 (3)
C7—N1—C10—C11	−162.7 (2)	C21—O3—C26—N2	45.1 (3)
C13—N1—C10—C11	11.3 (3)	C21—O3—C26—C25	158.96 (19)
O2—C10—C11—C12	91.7 (3)	C20—N2—C26—O3	−79.9 (3)
N1—C10—C11—C12	−28.2 (3)	C23—N2—C26—O3	105.5 (2)
C10—C11—C12—C13	35.7 (3)	C20—N2—C26—C25	162.3 (2)
C7—N1—C13—C12	−175.2 (2)	C23—N2—C26—C25	−12.4 (3)
C10—N1—C13—C12	10.8 (3)	C24—C25—C26—O3	−92.5 (3)
C11—C12—C13—N1	−28.2 (3)	C24—C25—C26—N2	26.8 (3)

### Hydrogen-bond geometry (Å, °)

**Table:**

*D*—H···*A*	*D*—H	H···*A*	*D*···*A*	*D*—H···*A*
C4—H4A···O1^i^	0.93	2.54	3.293 (4)	139
C16—H16A···O4^ii^	0.93	2.58	3.243 (3)	129

Symmetry codes: (i) −*x*, *y*−1/2, −*z*+1/2; (ii) *x*+1/2, −*y*+3/2, −*z*.
